# Effect of gene-lifestyle interaction on gestational diabetes risk

**DOI:** 10.18632/oncotarget.22999

**Published:** 2017-12-06

**Authors:** Polina V. Popova, Alexandra A. Klyushina, Lyudmila B. Vasilyeva, Alexandra S. Tkachuk, Yana A. Bolotko, Andrey S. Gerasimov, Evgenii A. Pustozerov, Ekaterina N. Kravchuk, Alexander Predeus, Anna A. Kostareva, Elena N. Grineva

**Affiliations:** ^1^ Almazov National Medical Research Centre, Saint Petersburg, Russia; ^2^ Department of Internal Diseases and Endocrinology, Pavlov First Saint Petersburg State Medical University, Saint Petersburg, Russia; ^3^ Saint Petersburg Electrotechnical University, Saint Petersburg, Russia; ^4^ ITMO University, Saint Petersburg, Russia; ^5^ Bioinformatics Institute, Saint Petersburg, Russia

**Keywords:** gestational diabetes mellitus, type 2 diabetes, lifestyle, single-nucleotide polymorphism, sausage consumption

## Abstract

We hypothesized that the association of certain lifestyle parameters with gestational diabetes mellitus (GDM) risk would depend on susceptibility loci. In total, 278 Russian women with GDM and 179 controls completed questionnaires about lifestyle habits (food consumption, physical activity and smoking). GDM was diagnosed according to the criteria of the International Association of Diabetes and Pregnancy Study Groups. Maternal blood was sampled for genotyping single-nucleotide polymorphisms (SNPs) in *MTNR1B* (rs10830963 and rs1387153), *GCK* (rs1799884), *KCNJ11* (rs5219), *IGF2BP2* (rs4402960), *TCF7L2* (rs7903146 and rs12255372), *CDKAL1* (rs7754840), *IRS1* (rs1801278) and *FTO* (rs9939609). Binary logistic regression revealed an interaction effect of sausage intake and the number of risk alleles of two SNPs (rs10830963 in *MTNR1B* and rs1799884 in *GCK*) on GDM risk (P < 0.001). Among women without risk alleles of these two SNPs, sausage consumption was positively associated with GDM risk (P trend = 0.045). This difference was not revealed in women carrying 1 or more risk alleles. The risk of GDM increased as the number of risk alles increased in participants with low and moderate sausage consumption (P trend <0.001 and 0.006, respectively), while the risk of GDM in women with high sausage consumption remained relatively high, independent of the number of risk alleles. These findings indicate that the association of sausage consumption with GDM risk can be determined based on the number of risk alleles of rs10830963 in *MTNR1B* and rs1799884 in *GCK*.

## INTRODUCTION

Gestational diabetes mellitus (GDM) is one of the most common disorders in pregnancy. Among the 15 centers that participated in the Hyperglycemia and Adverse Pregnancy Outcome Study, the prevalence of GDM was 17.8% (range 9.3-25.5%), as estimated with the new criteria of the International Association of Diabetes and Pregnancy Study Groups [1]. According to the International Diabetes Federation, the global prevalence of hyperglycemia in pregnancy is 16.9%, including total diabetes in pregnancy (known and previously undiagnosed diabetes) and gestational diabetes [2].

GDM is associated with significant short- and long-term adverse consequences for the mother and offspring, as it may necessitate caesarean delivery, cause birth trauma [3] and promote the future development of type 2 diabetes (T2D) [4]. There is increasing evidence that the intrauterine environment influences key developmental processes and long-term disease programming. More than 50 years ago, Y Pedersen formulated the famous theory that maternal hyperglycemia induces fetal hyperinsulinemia and thus causes adverse pregnancy outcomes [5]. In 1980, N Freinkel expanded the hypothesis of Pedersen by describing “fuel-mediated teratogenesis” [6]. He pointed out that excessive intake of nutrients causes fetal hyperinsulinemia and predisposes the fetus to hyperinsulinemia throughout life, leading to the development of obesity and diabetes. As many of the female offspring exposed to maternal diabetes during gestation develop obesity and diabetes (including GDM) by reproductive age, the consequences of overnutrition in utero may be viewed as a vicious cycle perpetuating for generations [7]. Thus, for the health of mothers and future generations, it is increasingly important to take preventive measures against GDM, detect GDM early, and determine the risk factors in GDM development.

T2D develops due to the interaction between genetic predisposition and lifestyle, as confirmed in a series of studies [8, 9]. The pathogenesis of GDM and T2D have many similarities. GDM is also assumed to result from the combination of genetic risk and an unfavorable lifestyle, as well as from “perinatal programming” caused by intrauterine overnutrition, as mentioned above (Pedersen-Freinkel hypothesis) [5, 6]. However, limited data support the hypothesis that gene-lifestyle interactions influence GDM development [10].

There are well-described non-modifiable risk factors for GDM, such as a history of GDM, a family history of T2D, and advanced maternal age [11, 12]. On the other hand, according to observational studies, modifiable factors such as unhealthy eating and a sedentary lifestyle are also associated with GDM risk [13]. However, meta-analyses have not yielded convincing data on how changes in modifiable factors (diet and lifestyle) affect GDM risk and adverse pregnancy outcomes [14]. This may be due to both the limitations of the studies analyzed and the different contributions of these factors to GDM development in women with different genetic predispositions.

A number of candidate gene studies have revealed the relationship of GDM with certain loci of genetic predisposition to T2D [15, 16] According to meta-analyses, variants in the following genetic loci are associated with an increased risk of GDM: melatonin receptor 1B *(MTNR1B)*, glucokinase (*GCK)*, transcription factor 7-like 2 (*TCF7L2)*, potassium inwardly rectifying channel, subfamily J, member 11 (*KCNJ11)*, regulatory subunit associated protein 1-like 1 *(CDKAL1)*, insulin-like growth factor 2 mRNA-binding protein 2 *(IGF2BP2)* and insulin receptor substrate 1 (*IRS1*) [16, 17] These findings have led to the study of gene-lifestyle interactions and their influence on GDM development. In a recent study by Grotenfelt et al., genetic variation in *MTNR1B* was found to modify the outcome of a lifestyle intervention for pregnant women with GDM [10]. However, the study included only high-risk individuals, and the sample size was small. Thus, further studies are needed to clarify whether other susceptibility loci influence the results of lifestyle interventions. Alongside lifestyle intervention trials, observational cohort studies can also be used to determine the interactions between genetic and lifestyle factors.

To the best of our knowledge, a standardized evaluation of the interactions between multiple susceptibility loci and lifestyle factors and their influence on GDM risk has not been published. Hence, we tested the hypothesis that the association of certain lifestyle parameters with the risk of developing GDM depends on susceptibility loci or combinations thereof. The following genetic variants were studied: rs10830963 and rs1387153 in *MTNR1B*, rs1799884 in *GCK*, rs5219 in *KCNJ11*, rs4402960 in *IGF2BP2*, rs7903146 and rs12255372 in *TCF7L2*, rs7754840 in *CDKAL1*, rs1801278 in *IRS1* and rs9939609 in fat mass and obesity-associated protein (*FTO)*.

## RESULTS

### Clinical characteristics of the participants

The clinical characteristics of the GDM patients and controls are shown in Table 1. The women with GDM were older and had a higher mean pre-pregnancy body mass index (BMI) than the controls. The patients with GDM more often had a history of arterial hypertension and GDM. Impaired glucose tolerance was also observed more frequently in the GDM patients, but the difference was not statistically significant (P = 0.052). No difference was observed in the frequency of polycystic ovary syndrome, the family history of diabetes or the multipara percentage between the groups. GDM patients had higher levels of triglycerides and very-low-density lipoprotein-cholesterol (VLDL-C) and lower levels of high-density lipoprotein-cholesterol (HDL-C) than control subjects. The percentage of large-for-gestational-age newborns was higher in the GDM group, although there was no difference between the groups in the birth weight or the frequency of delivery by caesarian section.

**Table 1:** Demographic and selected variables in GDM patients and controls

	GDM N=278	Control N=179	P
Age, years	31.8 ± 4.8	29.4 ± 4.8	<0.0001
Pre-pregnancy BMI, kg/m^2^	25.7 ± 5.9	22.9 ± 4.5	<0.0001
Family history of diabetes (%)	121 (43.5%)	70 (39.1%)	0.201
History of hypertension (%)	43 (15.5%)	12 (6.7%)	0.005
History of GDM (%)^*^	19 (13.8%)	0 (0%)	<0.001
History of IGT (%)	16 (5.8 %)	3 (1.7 %)	0.052
PCOS (%)	25 (9%)	10 (5.6%)	0.210
Parity:			
Nulliparae (%)	140 (50.4 %)	102 (57%)	0.099
Multiparae (%)	138 (49.6%)	77 (43%)	
Number of pregnancies^**^	2.1 ± 1.7	1.5 ± 1.4	<0.001
Total cholesterol (mmol/L)	6.68 ± 1.16	6.57 ± 1.34	0.374
Triglycerides (mmol/L)	2.26 ± 0.86	1.83 ± 0.63	<0.001
HDL-C (mmol/L)	1.93 ± 0.48	2.05 ± 0.44	0.013
VLDL-C (mmol/L)	1.05 ± 0.41	0.84 ± 0.29	<0.001
LDL-C (mmol/L)	3.69 ± 1.06	3.67 ± 1.19	0.844
Pregnancy outcomes^***^	N=257	N=158	
Delivery by caesarian section (%)	69 (26.8%)	33 (20.8%)	0.197
Neonatal birthweight, g	3471 ± 513	3444 ± 509	0.611
Percentage of LGA newborns (%)	40 (15.6%)	13 (8.2%)	0.034
Percentage of SGA newborns (%)	14 (5.4%)	12 (7.6%)	0.408

Note: BMI - body mass index, GDM - gestational diabetes mellitus, IGT - impaired glucose tolerance, PCOS - polycystic ovary syndrome, LGA - large for gestational age, SGA - small for gestational age.

^*^ - % counted for the number of multiparae (N=215).

^**^ - including the index pregnancy.

^***^ - pregnancy outcomes were available for a total of 415 women (257 GDM patients and 158 controls).

### Lifestyle patterns

**Figure 1:**
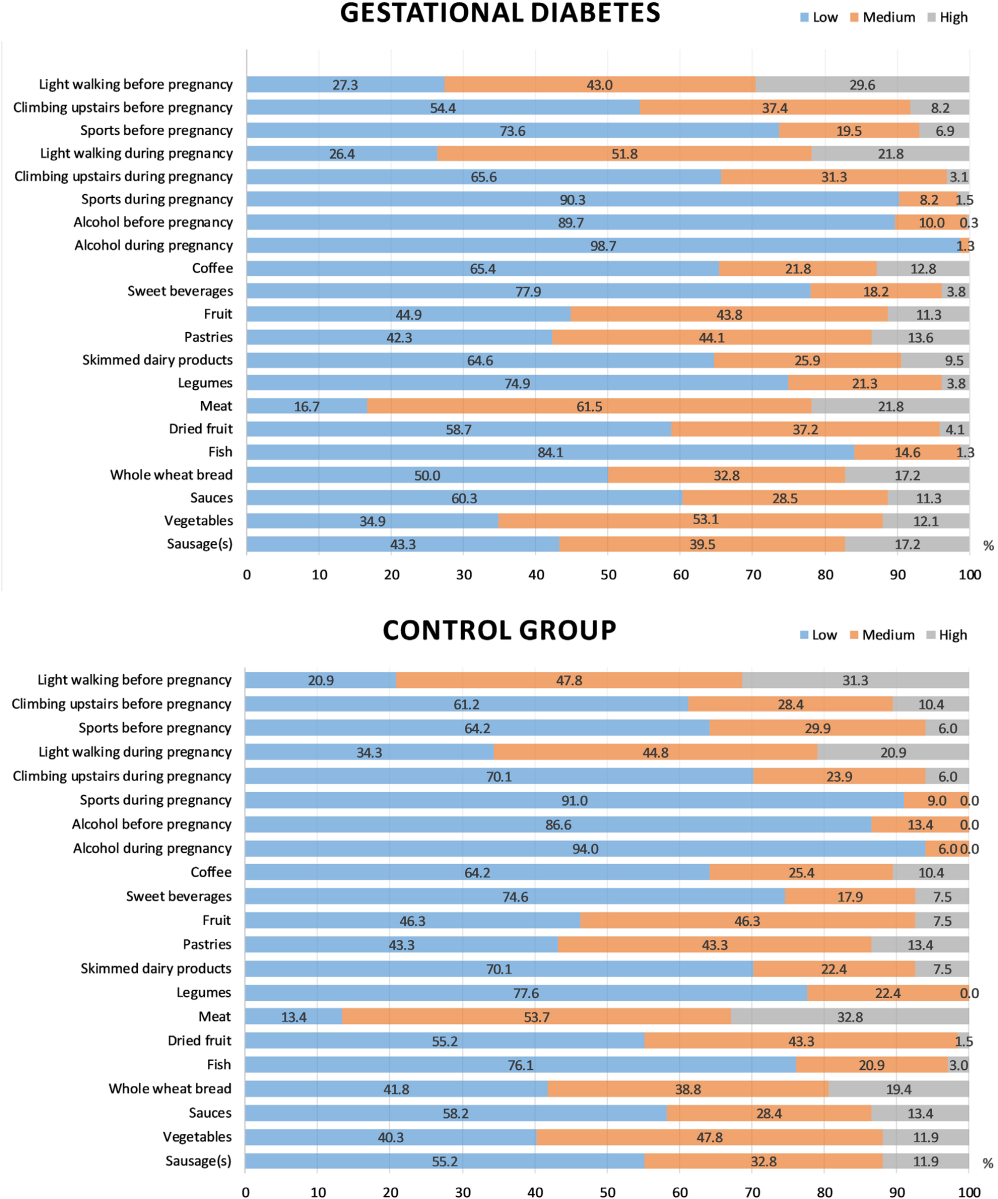
Lifestyle parameters in the GDM and control groups The bars of different colors reflect different frequency levels of consuming certain products and performing physical activity (low, medium and high). Depending on the factor, the following limits were selected: light walking, <30 min/day, 30-60 min/day, >60 min/day; climbing the stairs, <4/day, 4-16/day, >16/day; sports, <2 days/week, 2-3 days/week, >3 days/week; alcohol, <1/week, 1-3/week, >3/week; sweet beverages, <2/week, 2-4/week, >4/week; coffee, 0-1/day, 2-3/day, >3/day; fruits, <6/week, 6-12/week, >12/week; pastries, <2/week, 2-4/week, >4/week; skimmed dairy products, <3/week, 3-6/week, >6/week; legumes, <1/week, 1-2/week, >2/week; red and processed meats, <3/week, 3-6/week, >6/week; dried fruits and nuts, 0/week, 1-3/week, >3/week; fish, <3/week, 3-6/week, >6/week; whole-wheat bread, <1/week, 1-3/week, >3/week; sauces, <2/week, 2-4/week, >4/week; vegetables and salads, <6/week, 6-12/week, >12/week; sausages, <1/week, 1-3/week, >3/week.

We used a diet questionnaire to compare the frequency of consuming the main food groups, smoking and performing physical activity between the GDM and control groups. The results of the comparison are shown in Figure 1. Women with GDM did not consume legumes as often as those from the control group (P = 0.014). There was also a tendency to consume a higher amount of sausage and perform a smaller amount of physical activity (that is, climbing the stairs during pregnancy) among women with GDM (P = 0.101 and 0.073, respectively). Differences in other lifestyle parameters, however, were not identified.

In logistic regression analysis, high consumption of sausage before pregnancy (>3 times per week compared to less than once per week) was found to be associated with increased GDM risk (adjusted odds ratio [OR] = 2.2, 95% confidence interval [CI] = 1.2-4.1, P = 0.009) (Table 2). Legume consumption 1-2 times per week was associated with lower GDM risk than less frequent legume consumption (adjusted OR = 0.58, 95% CI = 0.36-0.94, P = 0.027). These associations remained after adjustments for age and pre-pregnancy BMI (Table 2).

**Table 2:** Association of significant lifestyle parameters with GDM risk

Lifestyle parameter	Frequency of practice	OR (95% CI)	P	OR (95% CI)^*^	P^*^
Consuming sausage(s)	<1/week (reference)	1		1	
	1-3/week	1.21 (0.80 – 1.82)	0.366	1.2 (0.8 – 1.8)	0.463
	>3/week	1.8 (1.04 – 3.23)	0.037	2.2 (1.2 – 4.1)	0.009
P trend			0.110		0.034
Consuming legumes	<1/week	1		1	
	1-2/week	0.63 (0.40 – 0.98)	0.042	0.58 (0.36 – 0.94)	0.027
	>2/week	3.90 (0.87 – 17.5)	0.076	4.16 (0.89 – 19.3)	0.069
P trend			0.02		0.012
Climbing stairs during pregnancy	<4/day	1		1	
	4-16/day	0.62 (0.41 – 0.94)	0.023	0.7 (0.4 – 1.0)	0.05
	>16/day	0.92 (0.33 – 2.61)	0.880	0.8 (0.3 – 2.3)	0.681
P trend			0.074		0.144
Pre-pregnancy physical activity score	1-3	1		1	
	4-6	0.56 (0.3 – 0.97)	0.041	0.65 (0.36 – 1.18)	0.161
	7-9	0.69 (0.3 – 1.45)	0.322	0.72 (0.33 – 1.56)	0.717
P trend			0.115		0.374

^*^Logistic regression analyses adjusted for age and pre-gestational BMI.

Having a moderate physical activity score before pregnancy and climbing the stairs during pregnancy were also associated with reduced GDM susceptibility, but these associations lost significance after adjustment for age and pre-pregnancy BMI (Table 2).

### Lipid profile and GDM risk

The association of GDM risk with fasting lipid levels at the time of an oral glucose tolerance test (OGTT) is shown in Table 3. The levels of triglycerides and VLDL-C were positively associated with GDM, and these associations remained significant after adjustment for age and pre-gestational BMI (OR [95% CI] = 2.03 [1.47-2.81] and 4.46 [2.22-8.93], respectively, P < 0.001 for both). The HDL-C level was inversely associated with the risk of GDM, but this association lost significance after adjustment for age and pre-gestational BMI (P = 0.165). GDM did not correlate with the levels of total cholesterol and LDL-C (data not shown).

**Table 3:** Association of lipid levels with GDM risk

	OR (95% CI)	P	OR (95% CI)^*^	P^*^
Triglycerides	2.32 (1.68 – 3.19)	<0.001	2.03 (1.47 – 2.81)	<0.001
HDL-C	0.58 (0.38 – 0.89)	0.014	0.72 (0.45 – 1.14)	0.165
VLDL-C	5.98 (3.01 – 11.89)	0.003	4.46 (2.22 – 8.93)	<0.001

^*^Logistic regression analyses adjusted for age and pre-gestational BMI.

### Genotypes

Table 4 presents the genotyping results and significant differences in the distribution of the rs10830963, rs1387153 and rs1799884 genotypes between GDM patients and controls. The genotype distributions of the studied single-nucleotide polymorphisms (SNPs) were all in Hardy-Weinberg equilibrium (P > 0.05). *FTO* rs9939609 was not associated with GDM in the total studied group, but the *FTO* genotype distribution differed significantly between GDM patients and controls in the subgroup of women with high sausage intake (>3/week) (P = 0.018).

**Table 4:** Genotype and allele distribution among GDM patients and controls

Gene	Variants	Minor allele	Genotypes in GDM patients (N=278), N (%)			Genotypes in controls (N=179), N (%)			P^*^
			AA	AB	BB	AA	AB	BB	
*MTNR1B*	rs10830963	G	49 (17.6)	133 (47.8)	96 (34.5)	17 (9.5)	69 (38.4)	93 (52.0)	0.001
	rs1387153	T	43 (15.5)	131 (47.1)	104 (37.4)	11 (6.1)	75 41.9)	93 (52.0)	0.001
*CDKAL1*	rs7754840	C	34 (12.2)	128 (46.0)	116 (41.7)	13 (7.3)	85 (47.5)	81 (45.3)	0.226
*GCK*	rs1799884	T	12 (4.3)	81 (29.1)	185 (66.5)	0 (0)	37 (20.7)	142 (79.3)	0.001
*IRS1*	rs1801278	T	0 (0)	21 (7.6)	257 (92.4)	0 (0)	19 (10.6)	160 (89.4)	0.309
*KCNJ11*	rs5219	T	54 (19.4)	122 (43.9)	102 (36.7)	31 (17.3)	92 (51.4)	56 (31.3)	0.288
*IGF2BP2*	rs4402960	T	24 (8.6)	134 (48.2)	120 (43.2)	26 (14.5)	76 (42.5)	77 (43.0)	0.120
*TCF7L2*	rs7903146	T	13 (4.7)	104 (37.4)	161 (57.9)	12 (6.7)	63 (35.2)	104 (58.1)	0.617
	rs12255372^**^	T	14 (5.1)	93 (33.8)	168 (61.1)	10 (5.7)	56 (31.8)	110 (62.5)	0.889
FTO^**^	rs9939609	A	60 (21.8)	136 (49.5)	79 (28.7)	28 (15.9)	87 (49.4)	61 (34.7)	0.208
FTO^***^	rs9939609	A	16 (31.4)	19 (37.3)	16 (31.4)	1 (4.5)	15 (68.2)	6 (27.3)	0.018

^*^ P value of two-sided chi-squared test for comparison of genotypes between the GDM and control groups. ^**^ - for this SNP, 451 women were genotyped (275 GDM patients and 176 controls), ^***^ - counted for the group of women with high sausage consumption (>3/week, N=73).

Logistic regression analysis confirmed the association of the G allele of rs10830963, the T allele of rs1387153 and the T allele of rs1799884 with increased GDM risk, which remained significant after adjustment for age and pre-pregnancy BMI (Table 5). However, the association of rs9939609 with GDM risk was not confirmed by logistic regression analysis, even in the subgroup of women with high sausage intake (P = 0.726).

**Table 5:** Association of three significant SNPs with GDM risk

Gene	Variant	Minor allele	OR (95% CI)	P	OR (95% CI)^*^	P^*^
*MTNR1B*	rs10830963	G	2.1 (1.4 – 3.0)	<0.001	2.0 (1.3 – 3.0)	0.001
	rs1387153	T	1.8 (1.2 – 2.6)	0.002	1.9 (2.3 – 3.9)	0.001
*GCK*	rs1799884	T	1.9 (1.2 – 3.0)	0.003	2.1 (1.3 – 3.3)	0.002

^*^Logistic regression analyses adjusted for age and pre-gestational BMI.

To test the independent effect of each SNP on GDM predisposition, we performed conditional logistic regression analyses. The effect of rs1387153 on GDM predisposition weakened after being conditioned by the other two SNPs. However, the effects of rs10830963 and rs1799884 remained significant after being conditioned by the other two SNPs (OR = 2.14, 95% CI = 1.09-4.18, P = 0.027, and OR = 2.01, 95% CI = 1.28-3.14, P = 0.002, respectively).

The influence of the combination of the two significant SNPs on GDM risk was also studied. The combination of the minor alleles of these two SNPs further increased the risk of GDM in a dose-dependent manner, compared with the absence of any risk alleles (P trend < 0.001) (Table 6).

**Table 6:** Cumulative effects of variant alleles rs10830963 and rs1799884 on GDM susceptibility

Number of risk alleles	GDM N (%)	Controls N (%)	OR (95% CI)	P	OR (95% CI)^*^	P^*^
0	55 (19.8)	78 (43.6)	1		1	
1-2	202 (72.7)	96 (53.6)	3.0 (2.0 –4.5)	<0.001	3.4 (2.1 –5.5)	<0.001
3-4	21 (7.6)	5 (2.8)	5.9 (2.1 – 16.7)	0.001	5.1 (1.7 – 15.3)	0.004
P trend				<0.001		<0.001

^*^Logistic regression analyses adjusted for age, pre-gestational BMI and the levels of triglycerides, HDL-C and VLDL-C.

Legend: Women with ‘0’ alleles carried no minor alleles of the two SNPs; women with ‘1-4’ alleles carried 1 to 4 variant alleles of the above-stated SNPs.

To determine whether the above-mentioned SNPs influenced GDM development by altering lipid levels, we compared the levels of serum total cholesterol, HDL-C, LDL-C, VLDL-C and triglycerides in women with and without the minor alleles of rs10830963, rs1387153 and rs1799884. No difference was found (data not shown).

### Gene-lifestyle interaction

We observed a significant interaction effect of the number of risk alleles of the two significant SNPs and the intake of sausage on the risk of developing GDM after adjustment for age, pre-gestational BMI and the levels of triglycerides, HDL-C and VLDL-C (P < 0.001) (Table 7). Among women without any risk alleles of the two SNPs, the level of sausage consumption was significantly positively associated with GDM risk (P trend = 0.045). This difference was not revealed in women carrying 1-2 or 3-4 risk alleles (P trend = 0.107 or 0.555, respectively). The number of risk alleles was positively associated with the OR for GDM risk in women with low and moderate levels of sausage consumption (P trend < 0.001 and 0.006, respectively), but not in women with a high level of sausage consumption (P trend = 0.223) (Table 7).

**Table 7:** Interaction effect of sausage intake and the number of the minor alleles of rs10830963 and rs1799884 on GDM risk (logistic regression analyses adjusted for age, pre-gestational BMI and the levels of triglycerides, HDL-C and VLDL-C)

Lifestyle parameter: frequency of practice	OR (95% CI)^*^			P trend	P inter-action
	Strata by the number of minor alleles of rs10830963 and rs1799884				
	0	1-2	3-4		
Sausage:					<0.001
<1/week	1	4.3 (2.0 – 9.2)	9.1 (1.7 – 49.5)	<0.001	
1-3/week	1.2 (0.5 – 3.1)	4.4 (1.9 – 9.6)	7.8 (1.4 – 43.5)	0.006	
>3/week	3.5 (1.2 – 10.1)	8.4 (3.0 – 23.4)	12.1 (0.8 – 57.2)	0.223	
P trend	0.045	0.107	0.555		

## DISCUSSION

Our study confirmed the association of three SNPs (rs10830963 and rs1387153 in *MTNR1B* and rs1799884 in *GCK*) with GDM risk, and identified several lifestyle parameters associated with an increased risk of GDM in Russian women. Two of these three SNPs (rs10830963 and rs1799884) remained significant after being conditioned by the other two SNPs. Moreover, we discovered that the number of minor alleles of these genes had an interaction effect with one of the lifestyle parameters (the frequency of sausage intake) on the development of GDM.

The risk allele (G) of rs10830963 in *MTNR1B* has been associated with the highest OR for GDM in several studies of Caucasian women [18, 19]. Furthermore, the interaction between this genetic variant and lifestyle intervention during pregnancy and its influence on the occurrence of GDM in high-risk women have recently been described [10]. The association of the T allele of rs1387153 in *MTNR1B* with GDM risk has also been described in several studies and meta-analyses [18, 20].

Melatonin, a hormone secreted by the pineal gland at night, governs the effects of the circadian rhythm on physiological functions (including glucose homeostasis) by binding to its receptors (MTNR1A and MTNR1B) [21]. *MTNR1B* is expressed in various cells and tissues, including the central nervous system and pancreatic beta cells [21]. The two genetic variants of *MTNR1B* described in this study are known to alter glucose metabolism by impairing early insulin secretion [22]. Melatonin has been shown to influence insulin secretion through several parallel signaling pathways in pancreatic beta cells [21]. Melatonin inactivates adenylate cyclase by binding to Gi-protein-coupled receptors, which in turn lowers cyclic adenosine monophosphate levels in the cell and reduces insulin secretion. Furthermore, melatonin inhibits the guanylate cyclase/cyclic guanosine monophosphate pathway and subsequently inhibits insulin secretion [21]. Both of these effects can elevate plasma glucose by reducing the secretion of insulin.

The association of rs1799884 in *GCK* with the risk of developing GDM has also been reported in several studies and meta-analyses [23]. GCK is the key enzyme in glucose phosphorylation and promotes the glucose-stimulated insulin secretion of beta cells in the pancreatic gland [24]. Inactivating mutations in the *GCK* gene are associated with the development of neonatal diabetes mellitus [25], while activating mutations are associated with hyperinsulinemia and hypoglycemia [26]. GDM may develop due to the combination of a genetic predisposition to impaired beta-cell function and an increase in insulin resistance as pregnancy progresses. As described above, both of the genes associated with GDM risk in this study (*MTNR1B* and *GCK*) are thought to modulate pancreatic islet beta-cell function.

Presumably, the interaction between multiple genetic and environmental factors determines the development of T2D and GDM. Here, we analyzed the relationship of a series of lifestyle parameters, including eating habits and physical activity, with GDM risk. We identified an interaction effect of genetic factors and the frequency of sausage intake on the risk of developing GDM. Although interaction effects of genes and particular lifestyle parameters on the risk of GDM have not been reported previously, several studies have described the association between diet and GDM development [27–29]. In our study, frequent sausage consumption (more than 3 times per week) before pregnancy increased the risk of developing GDM. Our results corroborate data demonstrating a positive association of the ‘Western’ diet, characterized by a high level of animal fat, with GDM development [28].

In the Nurses’ Health Study, Bowers et al. revealed that excessive intake of food rich in cholesterol and animal fat was associated with an increased risk of developing GDM [29]. In addition, they determined that replacement of calories from carbohydrates with a similar number of calories from fat was associated with a considerable increase in GDM risk [29]. Although the precise mechanisms whereby increased cholesterol and animal fat intake influence glucose homeostasis and GDM risk are not known, this association is plausible in view of physiology. An increased level of free fatty acids can suppress insulin-stimulated glucose uptake, thus contributing to the development of insulin resistance [30]. Moreover, the molecular mechanisms of lipid-induced insulin resistance have been described in several reviews [31, 32]. Increased tissue lipid levels and hepatic insulin resistance are linked through toxic lipid metabolites (diacylglycerol and ceramide species) that alter insulin signaling [31, 32]. Diacylglycerol activates protein kinase-C epsilon and thus reduces insulin-stimulated IRS2 phosphorylation and IRS2-associated phosphoinositide 3-kinase activity. Ceramide has been shown to inhibit the phosphorylation of AKT2 [32]. Consequently, diacylglycerol and ceramide impair the insulin-dependent activation of glycogen synthesis and suppression of gluconeogenesis.

Limiting the consumption of fatty foods (including sausage and sausage products) is one component of lifestyle modifications in GDM prevention programs. Our results support recent data from Grotenfelt et al. on the interaction between rs10830963 and lifestyle intervention, and its influence on the age-adjusted occurrence of GDM [10]. According to their study, the OR for GDM among women homozygous for the C allele of rs10830963 was significantly lower in the intervention group than in the control group (OR = 0.16, 95% CI = 0.03-0.85, P = 0.014). This difference was not detected in women carrying the G risk allele.

Although our study was observational, we identified a positive relationship between GDM risk and the frequency of sausage intake only in the group of women without risk alleles for GDM (including the G allele of rs10830963). We agree with the assumption of Grotenfelt et al. that this interaction may reflect an effect of the risk allele on insulin production. Indeed, polymorphisms in both genes associated with GDM in our study (*MTNR1B* and *GCK*) are known to alter insulin secretion [22, 24]. Sausage consumption probably promotes GDM development by increasing the levels of free fatty acids and lipid metabolites, thus contributing to insulin resistance. If impaired insulin production was the main reason for increased GDM risk among carriers of the risk alleles of *MTNR1B* and *GCK*, this may explain why carriers of the risk alleles did not benefit as much as non-carriers from insulin resistance reduction due to lower fat consumption. On the other hand, women without the risk alleles of *MTNR1B* and *GCK* still would have been prone to GDM development if they consumed high amounts of fat in the form of sausage, presumably due to increased insulin resistance.

Another explanation for this gene-lifestyle interaction might be that MTNR1B is involved in lipid metabolism. Ling et al. reported that *MTNR1B* variants were associated with the lipid profiles of a nonpregnant Chinese population. Though the mechanism underlying this association remains to be determined [33], melatonin is known to be important for lipid metabolism. Treatment with melatonin was found to improve the lipid profiles of type 2 diabetic patients [34] and diabetic rats by reducing triglyceride and LDL-C levels [35]. Consequently, the *MTNR1B* gene, which encodes a receptor for melatonin, may also be involved in lipid metabolism.

In our study, serum lipid levels obtained at the time of the OGTT were not associated with *MTNR1B* variants or the level of sausage consumption, but this may have been due to the limited sample size or significant dietary changes during pregnancy. Obese/overweight pregnant women or women with excessive weight gain during pregnancy are usually actively encouraged by gynecologists to restrict their caloric intake. A diet-induced reduction in lipid levels by the time of the OGTT (24^th^-28^th^ week of gestation) may have masked or attenuated differences in lipid levels that existed and induced insulin resistance before pregnancy or in the first trimester. On the other hand, our findings that third-trimester serum triglyceride and VLDL levels were positively associated with GDM risk are in line with the results of a recent meta-analysis demonstrating the association of lipid levels with GDM [36]. However, considering that the gene-lifestyle interaction remained significant after adjustment for lipid levels, we assume that increased lipid consumption can only partly explain this interaction, stressing the importance of impaired insulin secretion in the pathogenesis of GDM.

Given the association of GDM with sausage intake, we analyzed the possible association of GDM with *FTO* gene variant rs9939609, which is known to influence food behavior and lipid metabolism [37, 38]. *FTO* rs9939609 and adherence to the Mediterranean diet were reported to have a gene-diet interaction effect on T2D risk [38]. In our study, *FTO* rs9939609 variants were only associated with GDM risk in the subgroup of women with the highest level of sausage consumption, but not in the whole study population. Surprisingly, logistic regression analysis did not confirm the interaction effect of *FTO* rs9939609 variants and the frequency of sausage intake on GDM risk, probably due to the small sample size.

Since pregnancy is an important period for the primary prevention of diseases for the whole lifespan, our findings concerning the association of GDM risk with sausage and legume consumption may be helpful in clinical practice. A reduction of sausage intake to no more than 3 times per week and a moderate legume consumption of 1-2 times per week should be recommended during the nutritional counseling of pregnant women.

Another crucial factor determining GDM risk is physical activity. According to a meta-analysis of previous studies, the higher the level of physical activity before and during pregnancy, the lower the risk of GDM development [27]. Our data are consistent with this finding; however, the relationships identified in our study were weak and lost significance after adjustment for age and pre-gestational BMI, possibly due to the small sample size. In addition, we did not detect an interaction effect of physical activity and genetic factors on GDM risk.

The lack of interaction between genetic variants and physical activity scores or eating habits (except for sausage product consumption) in this study is in line with conclusions of the European Prospective Investigation into Cancer and Nutrition [28]. In this large multicenter cohort study, no significant interactions effects of genetic factors and physical activity or dietary habits on T2D risk were identified. This may be because the genes that interact with lifestyle factors differ from those known to predispose to T2D and GDM development. Another reason may be that participants tend to report their dietary habits inaccurately, thus attenuating associations that are likely to exist. This is a typical drawback of any epidemiological study assessing nutrition.

Our study had a few more limitations. Because of the cross-sectional study design, we could only assume but not prove that the lifestyle parameters before and during pregnancy were causally related to the risk of developing GDM. In addition, the responses of participants from different subgroups may have caused bias. For example, women who are overweight or gain excessive weight during pregnancy often underestimate their actual consumption of foods that are considered harmful. However, this possibility is difficult to calculate statistically. In addition, due to the relatively small number of women included in the study, the statistical power was low, leading to the wide range of CIs in the data analysis.

In conclusion, our results demonstrated that the association of sausage product consumption with GDM risk is determined by the number of risk alleles of rs10830963 in *MTNR1B* and rs1799884 in *GCK*. Both genes are implicated in pancreatic islet beta-cell function and glucose homeostasis. Possibly, the genetic predisposition due to the number of risk alleles and the adverse effects of lifestyle choices and food consumption are realized through different mechanisms, namely insulin secretion and tissue insulin resistance. Lifestyle modifications and related epigenetics may be more important determinants of GDM development in women without the risk alleles than in women with these alleles. Further studies are needed to clarify the influence of genetic factors on the effectiveness of lifestyle interventions to prevent GDM.

## MATERIALS AND METHODS

This case-control study was based on a study population of 1430 pregnant women screened for GDM at the National Almazov Medical Research Centre from January 2012 to December 2016. In total, 278 women with GDM and 179 controls were randomly selected from the cohort. The majority of cases and controls were ethnic Russians. Those who had pre-gestational diabetes, certain other diseases affecting carbohydrate metabolism, and fasting glucose levels >7.0 mmol/L were excluded. The study was approved by the ethical committee of the research center (protocol no. 119), and the participants provided written informed consent.

All the women were examined by an endocrinologist, who collected their medical histories and analyzed their medical charts. Medical history collection comprised the following data: arterial hypertension, GDM, impaired glucose tolerance, polycystic ovary syndrome, family history of diabetes, and parity.

The OGTT involved plasma glucose assessment at fasting, 1 h and 2 h after 75-g glucose intake during the 24^th^-28^th^ week of gestation. The venous plasma glucose concentration was determined by the glucose oxidase method. GDM was diagnosed according to the Russian national consensus [39] and the recommendations of the International Association of Diabetes and Pregnancy Study Groups (fasting glucose of ≥5.1 mmol/L, and/or ≥10.0 mmol/L after 1 h, and/or ≥8.5 mmol/L after 2 h) [40]. Pregnant women without diabetes were included as controls.

During the OGTT, maternal blood samples were obtained and stored at -80°C for further genotyping and serum lipid level assessment. Total cholesterol, HDL-C, LDL-C, VLDL-C and triglyceride levels in the fasting blood samples were measured through enzymatic colorimetric methods with a diagnostic reagent system for the Cobas Integra Autoanalyzer.

During the OGTT, the women were questioned about their clinical characteristics, and completed a special questionnaire under supervision. The questionnaire consisted of the following sections: frequency of consumption of basic products in a week (fruits, pastries, skimmed dairy products, legumes, meat, sausage products, dried fruits, fish, whole-grain bread, sauces, vegetables, alcohol, sweet drinks, and coffee), physical activity (walking duration in a day: <30 min/day, 30-60 min/day, or >60 min/day; frequency of climbing the stairs in a day: <4 flights/day, 4-16 flights/day, or >16 flights/day; frequency of sports activities: <2 days/week, 2-3 days/week, or >3 days/week), and smoking before and during pregnancy. The sections of the form were defined in a semi-quantitative way. This questionnaire has been previously reported [13, 41].

### DNA and genotyping

Genomic DNA was extracted from peripheral blood leukocytes with the FlexiGene DNA Kit (Qiagen, Hilden, Germany), following procedures recommended by the manufacturer. Genotyping was performed by real-time PCR with custom kits (Applied Biosystems, USA). The selection of SNPs was based on the results of recent meta-analyses confirming that variants within eight different genetic loci were associated with an increased risk of gestational diabetes: *MTNR1B* (rs10830963 and rs1387153), *GCK* (rs1799884), *KCNJ11* (rs5219), *IGF2BP2* (rs4402960), *TCF7L2* (rs7903146), *CDKAL1* (rs7754840) and *IRS1* (rs1801278) [16, 17]. A locus associated with food behavior and lipid metabolism in *FTO* (rs9939609) [37, 38] was added to the analysis, given the association of GDM with sausage intake.

### Statistical analyses

Data were statistically processed with SPSS 22.0 (SPSS Inc., USA). The data are presented as the mean ± standard deviation. The χ^2^ criterion was used to compare the distribution of qualitative characteristics. Differences in the quantitative characteristics of the groups were assessed with Student’s t-test. The differences were considered significant at P < 0.05.

Binary logistic regression (forward conditional) was performed to identify the contribution of factors such as lifestyle parameters, the above-mentioned SNPs, and their interaction to the risk of GDM. The age- and BMI-adjusted interaction effects of certain significant risk alleles and lifestyle parameters on GDM risk were assessed with logistic regression models, including the main effects of the genotypes and lifestyle parameters and their multiplicative terms.

As a dependent indicator, the presence or absence of GDM was determined. In total, 17 parameters were chosen as predictors of GDM: 11 parameters associated with the consumption of certain product groups (fruits, pastries, skimmed dairy products, legumes, meat, sausage products, dried fruits, fish, whole-grain bread, sauces, and vegetables), 3 parameters related to beverages (alcohol, sweet drinks, and coffee), and 3 parameters characterizing physical activity (walking, climbing the stairs, and performing sports). For each listed parameter, the intensity was estimated on an ordinal scale of three levels: low, medium and high. Smoking was marked as ‘yes’ or ‘no’. Smoking, alcohol intake and physical activity parameters were assessed separately before and during pregnancy. The level of physical activity was also evaluated according to the total number of points for each of the three activities before pregnancy (physical activity score 1) and during pregnancy (physical activity score 2).

## Abbreviations

GDM: gestational diabetes mellitus
T2D: type 2 diabetes
SNP: single-nucleotide polymorphism
BMI: body mass index
OR: odds ratio
CI: confidence interval
VLDL-C: very-low-density lipoprotein-cholesterol
HDL-C: high-density lipoprotein-cholesterol
